# A false alarm of COVID-19 pneumonia in lung cancer with anti-PD-1 related pneumonitis: a case report and review of the literature

**DOI:** 10.1186/s13256-020-02619-y

**Published:** 2021-02-01

**Authors:** Ying Dai, Sha Liu, Yiruo Zhang, Xiaoqiu Li, Zhiyan Zhao, Pingping Liu, Yingying Du

**Affiliations:** Division of Oncology, The First Affiliated Hospital of Anhui Medical University, 218 Jixi Road, Hefei, 230022 China

**Keywords:** COVID-19, Pneumonitis, Immunotherapy

## Abstract

**Background:**

Pneumonitis belongs to the fatal toxicities of anti-PD-1/PD-L1 treatments. Its diagnosis is based on immunotherapeutic histories, clinical symptoms, and the computed tomography (CT) imaging. The radiological features were typically ground-glass opacities, similar to CT presentation of 2019 Novel Coronavirus (COVID-19) pneumonia. Thus, clinicians are cautious in differential diagnosis especially in COVID-19 epidemic areas.

**Case presentation:**

Herein, we report a 67-year-old Han Chinese male patient presenting with dyspnea and normal body temperature on the 15th day of close contact with his son, who returned from Wuhan. He was diagnosed as advanced non-small cell lung cancer and developed pneumonitis post Sintilimab injection during COIVD-19 pandemic period. The chest CT indicated peripherally subpleural lattice opacities at the inferior right lung lobe and bilateral thoracic effusion. The swab samples were taken twice within 72 hours and real-time reverse-transcription polymerase-chain-reaction (RT-PCR) results were COVID-19 negative. The patient was thereafter treated with prednisolone and antibiotics for over 2 weeks. The suspicious lesion has almost absorbed according to CT imaging, consistent with prominently falling CRP level. The anti-PD-1 related pneumonitis mixed with bacterial infection was clinically diagnosed based on the laboratory and radiological evidences and good response to the prednisolone and antibiotics.

**Conclusion:**

The anti-PD-1 related pneumonitis and COVID-19 pneumonia possess similar clinical presentations and CT imaging features. Therefore, differential diagnosis depends on the epidemiological and immunotherapy histories, RT-PCR tests. The response to glucocorticoid is still controversial but helpful for the diagnosis.

## Background

Immune checkpoint blockade monoclonal antibodies have revolutionized anti-tumor treatments in advanced lung cancer [1]. Among the unique toxicity due to the immunotherapy, pneumonitis is the severe and fatal immune-related adverse event (irAE) [2], which is defined as noninfectious focal and diffuse inflammation of lung parenchyma [3]. The overall incidence ranges from 1 to 10% due to specific agents [4]. The diagnosis was based on the clinical symptom and exclusion of pneumonia and other pulmonary infections including coronavirus disease 2019 (COVID-2019). The typical features of CT imaging of COVID-2019 are multifocal bilateral ground glass opacities (GGOs) with patchy consolidations, distributed peripherally in sub pleural area of posterior part or lower lobes in lung. The diagnosis was fundamentally confirmed by positive real-time reverse-transcription-polymerase-chain-reaction (RT-PCR) results by respiratory or blood samples. Herein we report a COVID-2019 suspect case of one advanced lung cancer patient present with pneumonitis post sintilimab injection. The negative RT-PCR of coronavirus results and good response to prednisone has consolidated the diagnosis of anti-PD-1 related pneumonitis.

## Case presentation

**Fig. 1:**
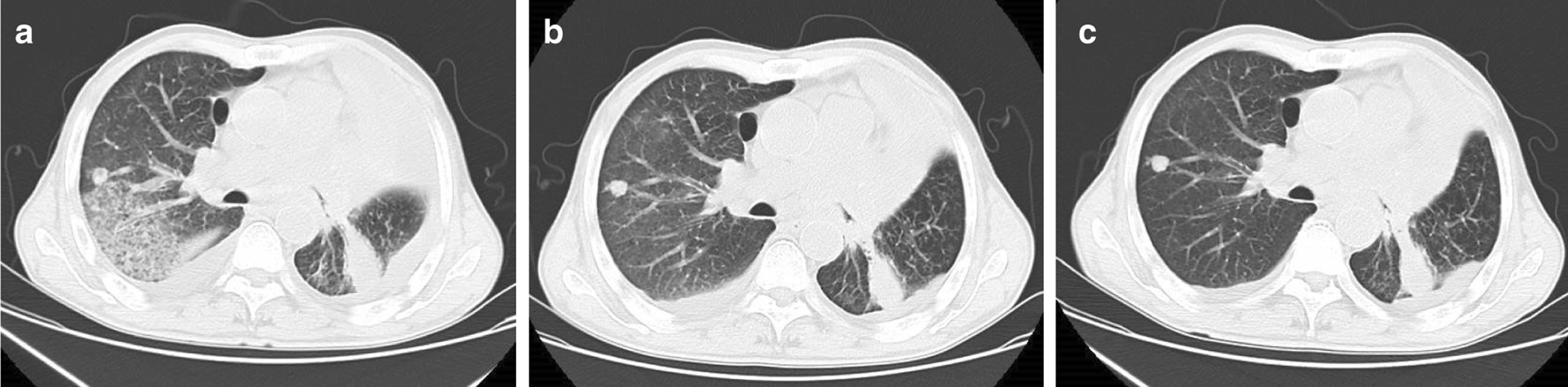
Assessment of the pneumonitis lesion via chest CT scan before (**a**) and after 3 days (**b**) and 7 days (**c**) of prednisolone and antibiotics treatment

A 67-year-old Han Chinese male smoker present with nonproductive cough and increasing shortness of breathless. The chest CT imaging showed central lung cancer located in left lobe, accompanied with pulmonary artery invasion, obstructive atelectasis and pleural effusion. The biopsied pathology from bronchoscopy indicated squamous cell carcinoma. The cytology from pleural effusion showed positive tumor cells. The patient was finally diagnosed as metastatic lung cancer squamous cell carcinoma. He was injected with 10 cycles of sintilimab, concurrent with chemotherapy containing gemcitabine and carboplatin in the first 4 cycles. The immunotherapy was replaced by paclitaxel for one cycle when CT evaluation suggested progressed disease. The dyspnea appeared on the 15th day of close contact with his son, who returned from Wuhan but not accompanied with fever. The relevant physical examinations included rales of lung and low breath sound of the left thorax. The chest CT (Fig. 1A) indicated peripherally subpleural lattice opacities at the inferior right lung lobe and bilateral thoracic infusion. The complete blood count showed increased white blood cell (WBC) and neutrophilic granulocyte with concurrently decreased lymphocyte. The C reaction protein (CRP) level was 97.68 mg/L, but procalcitonin was normal. As suspect of COVID-19 infection, the patient was treated in an isolation ward, and the double RT-PCR results from swab samples within 72 hours remained negative. No pathogen was cultured from sputum samples. The patient was treated the daily dose of 80 mg prednisolone and meropenem for 7 days. On the 3rd-day post treatment, the chest CT (Fig. 1b) showed an attenuated inflammatory lesion. The daily dose of prednisolone was stepwise reduced to 40 mg for 7 days and then minimally 20 mg. Secondary to 7 days of piperacillin tazobactam injection, the chest CT (Fig. 1c) demonstrated the former lesion almost absorbed, in line with prominently falling CRP level to 22.17 mg/L. The anti-PD-1 related pneumonitis with bacterial infection was finally diagnosed based on the clinical evidence and good response to the prednisolone and antibiotics. Due to continued hemoptysis, the patient started with afatinib and stable disease was evaluated by CT imaging. He died post one month of oral treatment.

## Discussion and conclusion

Pneumonitis can develop at any time once the immunotherapy initiated. The median onset of pneumonitis is in average three months post the latest immunotherapy [5]. In our case, the first onset of pneumonitis arose after two months of the last cycle of sintilimab. The diagnosis mainly relies on the CT images, infectious disease (ID) consult, and pulmonary consult. The bronchoscopy is also alternative if required [6]. According to the CT presentation, the pneumonitis is more extensive in the lower lobes instead of middle and upper lobes. The radiographic features vary, but the majority displays GGOs in cryptogenic organizing pneumonia (COP) pattern. Thus, it is difficult to differentiate immunotherapy associated pneumonitis and COVID-19 pneumonia sole by CT imaging.

To diagnose COVID-2019 pneumonia, the positive real time RT-PCR assay result is fundamental. However, the first task is to confirm Wuhan exposure history or close contact with people from Wuhan or COVID-19 patients in the last 2 weeks according to China updated the novel coronavirus pneumonia diagnosis and treatment program (trial version) [6, 7]. Other criteria include fever and/or respiratory symptoms, imaging evidence of viral pneumonia, and normal or low white blood cell count or lymphopenia.

In our case, the hypoxemia firstly appeared at the 15th day of close contact on his son, who has a residential history in Wuhan. The son was still suspicious of asymptomatic COVID-19 infection even with normal chest CT imaging, and the complete blood cell count test, a s his coronavirus status was not tested when his father was admitted. The following negative RT-PCR tests from the patient and a good response to prednisolone indirectly supported the diagnosis of anti-PD-1 related pneumonitis. Additionally, the son’s RT-PCR test in May was also negative, which consolidated the conclusion mentioned above.

Our patient started on one cycle of paclitaxel followed by progression on a CT scan and developed dyspnea within 5 weeks. It is important to exclude taxane-induced pulmonary toxicity. As it is rarely reported in clinical trials and case reports, the overall occurrence is difficult to assess. The high-risk factors included a history of lung pathology such as emphysema or interstitial lung disease (ILD). Typical CT imaging reflected diffuse bilateral process, such as GGO [8]. In our case, the pneumonitis presented with lattice opacities limited in unilateral lung. Thus, the prior diagnosis of taxane-induced pneumonitis was not considered for this patient.

The pneumonitis symptoms are easily ignored as they are masked by the primary lung cancer disease such as large tumor mass. A thorough evaluation is encouraged for clinicians in case of suspicious onset including dyspnea, cough and fever. Bronchoscopy with bronchoalveolar lavage (BAL) is recommended in early stage to rule out alternative diagnosis such as infectious pneumonia. However, in COVID-19 pandemic period, the endoscope was not encouraged in case of crossover infection. According to the blood test and CT imaging, the bacterial infection of our patient was at least not excluded and the comorbid of pneumonitis was more likely to occur.

Steroid therapy is still controversial in the management of COVID-19 pneumonia whereas it promptly relieves respiratory symptoms in anti-PD-1 related pneumonitis. In pandemic areas, the anti-PD-1 related pneumonitis was to some extent masked by coronavirus pneumonia due to the alert of COVID-19, leading to prolonged diagnosis and delayed treatments. Therefore, the routine screening of COVID-19 has been mandatory before hospitalization in China. In conclusion, the differential diagnosis should fundamentally base on the epidemiological information, RT-PCR tests and past immunotherapeutic history.

## Data Availability

All data generated or analyzed during this study are included in this published article.

## Abbreviations

BAL: Bronchoalveolar lavage
CT: Computed tomography
CRP: C reaction protein
COP: Cryptogenic organizing pneumonia
COVID-19: 2019 Novel Coronavirus
irAE: Immune-related adverse event
ID: Infectious disease
ILD: Interstitial lung disease
GGOs: Ground glass opacities
RT-PCR: The real-time reverse-transcription polymerase-chain-reaction
WBC: White blood cell
